# A Caution against Septic Germicides

**Published:** 1887-01-01

**Authors:** J. McF. Gaston

**Affiliations:** Professor of Surgery in the Southern Medical Ccollege


### Southern Medical Record.

A MONTHLY JOURNAL OF PRACTICAL MEDICINE.

☞All Communications must be addressed to R. C. Word, M. D., Managing Editor.

"*Quicquid Præcipies Esto Brevis.*"

Vol. XVII. ATLANTA, GA., JANUARY 1, 1887. No. 1.

### ORIGINAL AND SELECTED ARTICLES.

### A CAUTION AGAINST SEPTIC GERMICIDES.

By J. McF. Gaston, M. D.,

Professor of Surgery in the Southern Medical Ccollege.

REPORTED FOR THE SOUTHERN MEDICAL RECORD.

In a discussion upon "The Antiseptic Method in Surgery," at a meeting of the Atlanta Society of Medicine on Tuesday evening, December 7th, Dr. J. McF. Gaston took a position of independent conservatism in opposing the reckless employment of that class of germicides that are calculated to do injury to the patient by their absorption.

He stated that all parties were agreed upon the fundamental principle of the so-called antiseptic method in excluding, as far as practicable, the entrance of air into wounds, but that it was not desirable under all circumstances to close up a wound, and it was very necessary to have drainage from such as were threatened with disintegrating processes, so that the open treatment of some wounds fulfilled best the antiseptic indications.

Those who followed most rigidly the precepts of Lister, or adopted the more recent advances in aseptic precautions, cannot always secure the deep-seated structures against changes that result in disorganization; the cutaneous covering may unite by adhesive inflammation, and shut up the disorder within, until suppuration is developed, and demands an outlet by reopening the line of union in the skin. That the injurious contact of the atmosphere depended upon the presence of morbific germs was alleged by those who considered that all the ills that flesh is heir to were due to bacteria, micrococci, spirilia and bacilli, with their outgrowth of spores, etc. But it was by no means a proof of the causative operation of microbes that they were discovered by the microscopist in the progress of different diseases; and while the contagious disorders were traceable to distinct organisms, and their origin in like manner, as scabies to the animalcula producing it, there is not sufficient evidence of thier relation as concomitants of other diseases to exclude them from the category of effects or consequences of the abnormal condition. As the larvæ deposited by the fly upon a sore, and developed subsequently in the form of maggots, are simply an accident attending its existence, and not in any way concerned in its production, so in like manner the presence of the various micro-organisms which are discovered by the microscopist may not originate the different diseases with which they are associated. The special manifestations of microbial development corresponding to distinct forms of disease, such as the comma bacillus in cholera, the tubercle bacillus, and even the recently verified microbe of malaria, which has been described by Dr. William Osler in common with others, only indicates that each has its form corresponding to its habitat, and leads to no definite conclusion in regard to its causative influence. The germicides may hence be adapted to eradicate the microbes, and yet not tend to cure the disease associated with it.

In case there is present any degeneration of the structures calling for curatives, there can be no doubt as to the application of such remedies as are likely to oust the septic influence, and he was disposed to adopt the most vigorous aseptic means as corrective of the disorder.

Pyæmia and septicæmia may doubtless be arrested by the timely use of antiseptic washes, or other local applications to the surface involved. The former, resulting from the absorption of pus into the circulation, and latter associated with a vital degeneration of parts independent of suppuration, may ensue from circumscribed modifications in the tissues that very frequently demand measures of this class to be applied vigorously and promptly for their correction. In this view of antiseptic treatment, it was certainly good surgery to adopt the most energetic means at our command. But the prime consideration of the surgeon is to make sure of doing no harm to his patient, and the use of carbolic acid and bi-chloride of mercury as prophylactics is attended with such risk of injurious effects upon the general organization as to induce caution in their promiscuous application. The cases of renal trouble from the former, and of intestinal disorder from the latter, terminating fatally in some instances, are well calculated to deter the circumspect operator from applying these potent agents to freshly cut surfaces in connection with extensive operations.

Be sure you do no harm should ring in the ears of the surgeon at every step of an important operation upon vital structures, and the desire to kill germs can never afford an excuse for jeopardizing the safety of his patient by the absorption of poisonous substances which may be classified as antiseptics. We know to a certainty that serious consequences have followed the application of bi-chloride of mercury, and the injury resulting from its use in numerous instances cannot be compensated by any supposed virtues it may possess as a germicide; and it should be excluded entirely from the antiseptic method on account of its recognized septic and even deadly influence upon the tissues of the body.

It is not a question of indifference as to the effect of such a poison upon the organization, but a positive evil influence is so likely to result that no prudent surgeon can be justified in exposing his patient to such imminent danger, when there is no commensurate advantage from this risk of an unnecessary treatment.

That this so-called antiseptic method is not requisite for the successful management of surgical cases is abundantly shown by the superior results attained by surgeons who have ignored the theory and disregarded the practice in their operations, after having previously tested and abandoned the antiseptic proceeding.

Lawson Tait, Thomas Keith and George Granville Bantock, have operated of late without observing antiseptic methods, or using antiseptics of any kind in their dressings, with better results in abdominal surgery, than has accompanied the operations of any other surgeons throughout the world who have adhered to all the details of antiseptics. If suceess, as claimed in these instances, has been secured without using the antiseptic method at all, it is fair to infer that the benefits alleged to flow from its adoption by others may be attributed to other agencies, and doubtless the element of most efficacy apart from the skill of the operator is great attention to cleanliness.

While the hypothesis upon which Sir Joseph Lister proceeded may have led to an erroneous theory of diseases, the great practical outgrowth of his views in getting rid of filth in every shape and form, as a preliminary measure of surgical treatment, has redounded to the success of operations in every department of surgery throughout the civilized world, and we all owe him a great debt of gratitude.

But there were hurtful features connected with the original employment of carbolic acid spray, which proved injurious to patient and operator with all the attendants; and Keith boldly raised his voice against it in the meeting of the International Congress of 1881, in London, so that now it is seldom used anywhere.

If all the good features of Listerism were adopted and the bad elements completely eliminated, while cleanliness is carried out absolutely, we ought to attain the most satisfactory results. But in the view of many advocates of the antiseptic method in surgery, germicides have been held to be of paramount importance in the prophylactic appliances; and it is evident that the normal state of the tissues presented in most operations, does not call for the application of this class of agents, but on the contrary must be unfavorably affected by being brought in contact with even very weak solutions of corrosive sublimate.

It is therefore held and insisted on that a resort to such poisonous substances as germicides, while effectual in destroying microbial developments, are prejudicial in many instances to the integrity of the vital structures to which they are applied, and by their absorption prove destructive to the life of the patient. That they are unneccessary is clear from the best results being secured without their use, and the liability to injurious effects is so well established in the reports of serious results, that it is incumbent upon every surgeon to weigh in the scale of reason and experience the advantages and the disadvantages of the antiseptic method in surgery, as practiced at present.

